# ACCORD guideline for reporting consensus-based methods in biomedical research and clinical practice: a study protocol

**DOI:** 10.1186/s41073-022-00122-0

**Published:** 2022-06-07

**Authors:** William T. Gattrell, Amrit Pali Hungin, Amy Price, Christopher C. Winchester, David Tovey, Ellen L. Hughes, Esther J. van Zuuren, Keith Goldman, Patricia Logullo, Robert Matheis, Niall Harrison

**Affiliations:** 1 Global Medical Affairs, Ipsen, Abingdon, UK; 2 Faculty of Medical Sciences, Newcastle University, Newcastle, UK; 3 Stanford Anesthesia, Informatics and Media Lab, Stanford University School of Medicine, Stanford, CA USA; 4 Oxford PharmaGenesis, Tubney, Oxford, UK; 5 Journal of Clinical Epidemiology, Sussex, UK; 6 Sciwright Limited, Somerset, UK; 7 Leiden University Medical Centre, Leiden, The Netherlands; 8 Global Medical Affairs, AbbVie, North Chicago, IL USA; 9 Centre for Statistics in Medicine (CSM), University of Oxford, and EQUATOR Network UK Centre, Oxford, UK; 10 International Society for Medical Publication Professionals, New York, NY USA; 11 Ogilvy Health, London, UK

**Keywords:** Methodology, Guidelines, Reporting quality, Reporting completeness, Checklist, Delphi technique, Consensus, Nominal group technique, Consensus development conference

## Abstract

**Background:**

Structured, systematic methods to formulate consensus recommendations, such as the Delphi process or nominal group technique, among others, provide the opportunity to harness the knowledge of experts to support clinical decision making in areas of uncertainty. They are widely used in biomedical research, in particular where disease characteristics or resource limitations mean that high-quality evidence generation is difficult. However, poor reporting of methods used to reach a consensus – for example, not clearly explaining the definition of consensus, or not stating how consensus group panellists were selected – can potentially undermine confidence in this type of research and hinder reproducibility. Our objective is therefore to systematically develop a reporting guideline to help the biomedical research and clinical practice community describe the methods or techniques used to reach consensus in a complete, transparent, and consistent manner.

**Methods:**

The ACCORD (ACcurate COnsensus Reporting Document) project will take place in five stages and follow the EQUATOR Network guidance for the development of reporting guidelines. In Stage 1, a multidisciplinary Steering Committee has been established to lead and coordinate the guideline development process. In Stage 2, a systematic literature review will identify evidence on the quality of the reporting of consensus methodology, to obtain potential items for a reporting checklist. In Stage 3, Delphi methodology will be used to reach consensus regarding the checklist items, first among the Steering Committee, and then among a broader Delphi panel comprising participants with a range of expertise, including patient representatives. In Stage 4, the reporting guideline will be finalised in a consensus meeting, along with the production of an Explanation and Elaboration (E&E) document. In Stage 5, we plan to publish the reporting guideline and E&E document in open-access journals, supported by presentations at appropriate events. Dissemination of the reporting guideline, including a website linked to social media channels, is crucial for the document to be implemented in practice.

**Discussion:**

The ACCORD reporting guideline will provide a set of minimum items that should be reported about methods used to achieve consensus, including approaches ranging from simple unstructured opinion gatherings to highly structured processes.

**Supplementary Information:**

The online version contains supplementary material available at 10.1186/s41073-022-00122-0.

## Background

Evidence-based medicine relies on three factors: current best evidence based on clinical and real-world studies, individual clinical expertise, and the desires of the patient [1]. Clinical data gathered from systematic reviews, high-quality randomised clinical trials, and observational studies have complementary roles in generating robust evidence [2, 3]. However, healthcare providers face difficult treatment decisions if the available information on a subject is inadequate, contradictory, limited, or does not exist.

The COVID-19 pandemic has brought this situation of lack of evidence into stark relief, as crucial decisions have to be made during any rapidly emerging public health crisis [4]. However, there are areas of medicine for which high-quality evidence generation can be difficult. This is due to disease characteristics such as rare occurrence and clinical heterogeneity among patients with the same condition, which can mean either that trials are difficult to interpret or that they may only be directly applicable to a subset of patients [5, 6]. A lack of resources and/or infrastructure can also be limiting [6, 7]. Moreover, even when evidence does exist, in medical situations with multiple considerations or confounding factors, there is the need to prioritise the use of available evidence to optimise outcomes [8].

Therefore, when no robust evidence is available, when divergent guidance exists, or when there is a need for collective judgement to increase reliability and validity, guidelines for clinical decision making or methodological or reporting approaches may be formulated based on expert consensus only [9–11]. Consensus methods provide opportunities to harness the knowledge of experts to support clinical decision making in areas of uncertainty [12]. As with all studies, appropriate methods and transparent reporting are key; however, the method used to reach consensus is not always clearly reported [11, 13].

Multiple methods are used to develop consensus-based publications. These range in methodological rigour from informal “expert consensus meetings” to structured or systematic approaches such as the Delphi method and the nominal group technique (NGT). Both Delphi and NGT are used for generating ideas or determining priorities, aiming to achieve general convergence, usually through voting on a series of multiple-choice questions [14–17]. In Delphi, and more recently electronic Delphi (eDelphi), individuals vote anonymously, while NGT is usually face-to-face [8, 18, 19]. The techniques and methodological steps used to reach consensus can vary (Table 1).

**Table 1:** Possible types of consensus methods and characteristics that can be mixed or used separately in different stages of studies to reach consensus

Method	Characteristics	Data analysis
Consensus conference or meeting [20–22]	Face-to-face meetings where a group of participants, usually experts in one field of knowledge, discuss one or more topics, prompted by facilitators, and have to either create ideas/statements or decide/vote on pre-set topics/statements. The discussion is frequently prompted by evidence from the literature — or the lack of it.	Qualitative or quantitative, or mixed.
Nominal group technique (NGT) [20, 22, 23]	As in conference meetings, in NGT, face-to-face meetings are held, but several sessions are organised with iterative stages. In the first step, suggestions are collected from the groups into questionnaires or lists of topics circulated again in the second step. In the second stage, participants need to vote or rate, usually using scales (like Likert scales). The group then discusses the aggregated summary of the voting or rating. The group is not anonymous and may include experts and non-experts. A facilitator makes sure every participant is given the opportunity to speak and vote.	Qualitative initially and then quantitative when responses are aggregated and summarised.
Delphi [12, 20, 22–30]	The three principles of the Delphi technique are: 1) anonymity during voting/selecting/rating (participants do not meet); 2) multiple rounds (at least 2) and 3) feedback to participants to inform them about each last voting/rating before they start the next round. Delphi was traditionally organised by postal mail in the past, and now electronic specialised survey platforms facilitate the process.	Quantitative for voting/rating, qualitative when extra comments/suggestions are allowed.
Other mixed methods [20, 22]	A consensus study can begin with simple focus groups to collect ideas, stories, experiences, and general opinions to start a more structured NGT or Delphi exercise. Frequently, two or more methods are used. For example, a Delphi activity can be used initially with the list of statements approved to be discussed in consensus conferences where final decisions are made, sometimes referred to as a “modified Delphi”.	Qualitative methods are used when perceptions, stories, and experiences are collected. Several quantitative statistics can be used to summarise voting and ratings.

In group decisions, a wider range of knowledge may be drawn upon, the interaction between group members can stimulate and challenge received ideas, and idiosyncrasies may be filtered out through the group prioritisation process [19, 31–33]. The use of structured, systematic approaches to reach consensus is supported by the observation that, in an unstructured group meeting, there is the risk of a single individual dominating the discussion and decisions may be portrayed as unanimous when, in reality, there is dissent within the group [31]. Even within structured consensus meetings, depending on their roles, a few panel members can dominate the discussion [34]. Furthermore, individuals may be unwilling to retract long-held views in open discussion. For these reasons, structured approaches including a step where responses are anonymised are generally held to be superior to unstructured methods to achieve consensus [35, 36].

Developing consensus-based publications using robust methods is vital, but poor execution or reporting can render the techniques used for gathering opinion susceptible to criticism [37–40]. To take one of the most widely-used and most rigorous consensus methodologies, the Delphi method has been used extensively in a wide range of sectors including military, education, social science and healthcare since its conception in the 1950s at the RAND Corporation [41]. This is because it has the potential to mitigate many of the aforementioned pitfalls in group decisions, such as the risk of peer pressure in techniques such as the NGT [38, 42]. Due to its versatility, the Delphi method can be modified to meet individual study needs. However, the reporting of such “modified Delphi” methods may lack clarity on the details of the process involved or the rationale for the modification [38, 42].

Definitions of the thresholds for consensus (i.e., approval rates), for example, can vary or be poorly described in studies using consensus [43]. Other reporting or methodological problems identified are that analytical methods may not be predefined [37, 43], the recruitment process used to identify the experts may not be explicit [44], or the funding source not clearly disclosed [45]. In fact, critics suggest the term “Delphi research” be phased out in academic publications to force authors to more precisely describe the methodology used [46].

**Fig. 1:**
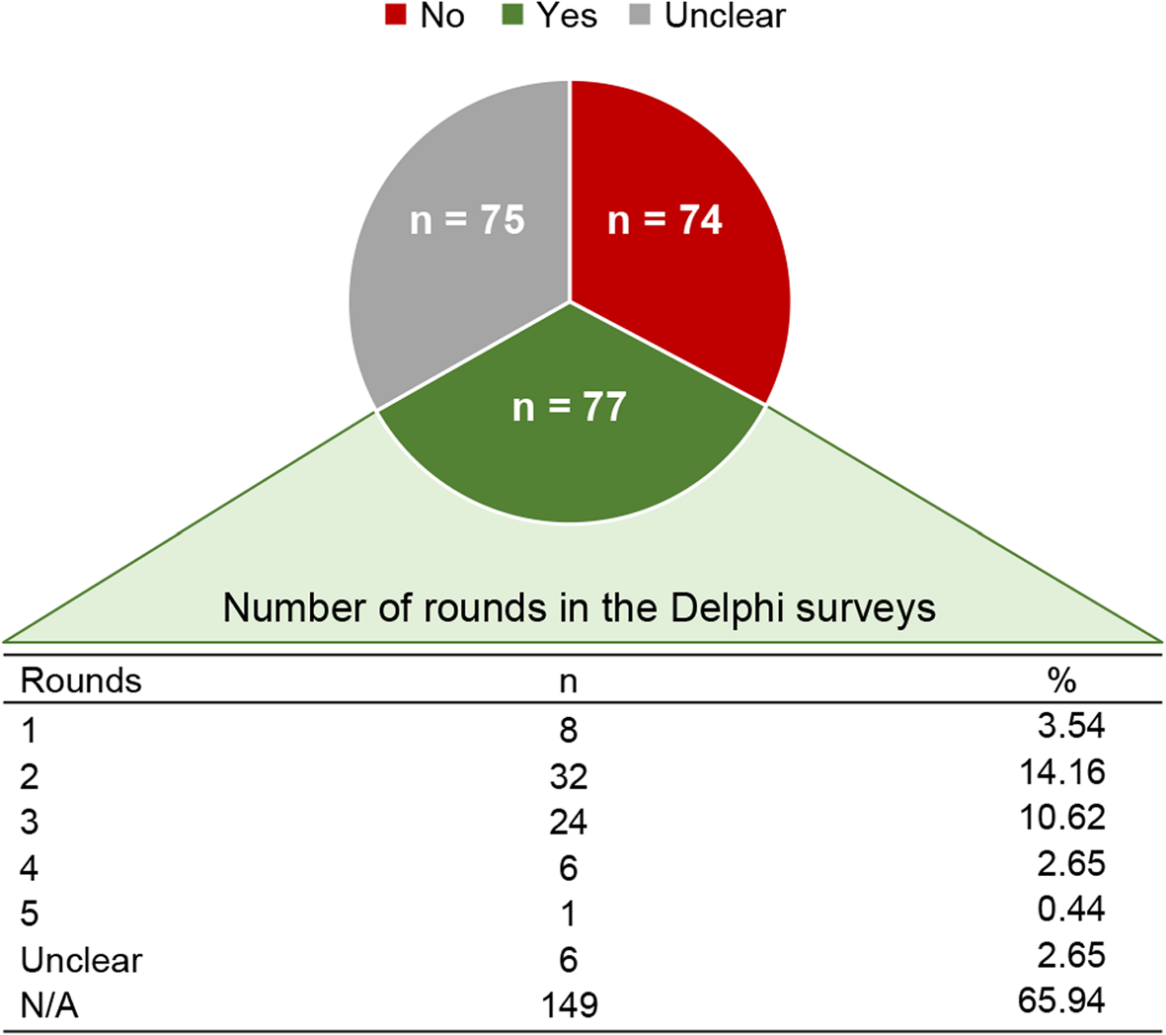
Methodology declared by authors in developing a reporting guideline added to the EQUATOR database from inception to October 2018 ( N = 226)

The lack of appropriate and transparent description in publications of the consensus methods used suggests that a reporting guideline is needed. A reporting guideline comprises “a checklist, flow diagram, or explicit text to guide authors in reporting a specific type of research, developed using explicit methodology” [11]. Consensus methods themselves play an important role in the development of reporting guidelines in various fields of health. As part of an ongoing audit of the EQUATOR database [47], it has been observed that, of the 226 reporting guidelines added between database inception and October 2018, only one third (77/226) explicitly mentioned the use of Delphi methodology (Fig. 1), while in another third (75/226), the information was not reported. A systematic review of the EQUATOR database indicated a similar result and added that among the reporting guidelines that mentioned the Delphi method, the description of details of the participants, number of rounds, criteria for dropping items or stopping the rounds was not always reproducible [48].

A range of methods can be used to reach consensus for clinical guidance, nomenclature, and other approaches in healthcare and public health [49]. However, to the best of our knowledge, the only reporting guidance in healthcare using consensus research is the CREDES (guidance on Conducting and REporting DElphi Studies) Statement, which provides valuable recommendations for the reporting of Delphi consensus in palliative care [38]. Nevertheless, CREDES is specific to palliative care and is limited to the Delphi method [38], which leaves a gap for a reporting guideline that can be applied to other biomedical areas and consensus processes involving non-Delphi based methods or “modified Delphi” — an issue that CREDES acknowledges. Moreover, CREDES does not provide a detailed checklist to guide the incorporation of essential steps to be reported.

Detail-oriented reporting can help readers of publications to understand the key elements of the process – the methodology used, the participants involved, and how the study was conducted including the criteria for statement approval. Our objective is therefore to systematically develop a reporting guideline to help the biomedical research and clinical practice community describe the methods used to reach consensus in a complete, transparent, and consistent manner. Our aim is that the reporting guideline is appropriate to describe all types of consensus methodology. The reporting guideline for consensus-based biomedical publications will include a general statement with a checklist and an explanation and elaboration (E&E) document, including examples of good reporting. It will be identified under the acronym ACCORD (ACcurate COnsensus Reporting Document).

## Methods/design

**Fig. 2:**
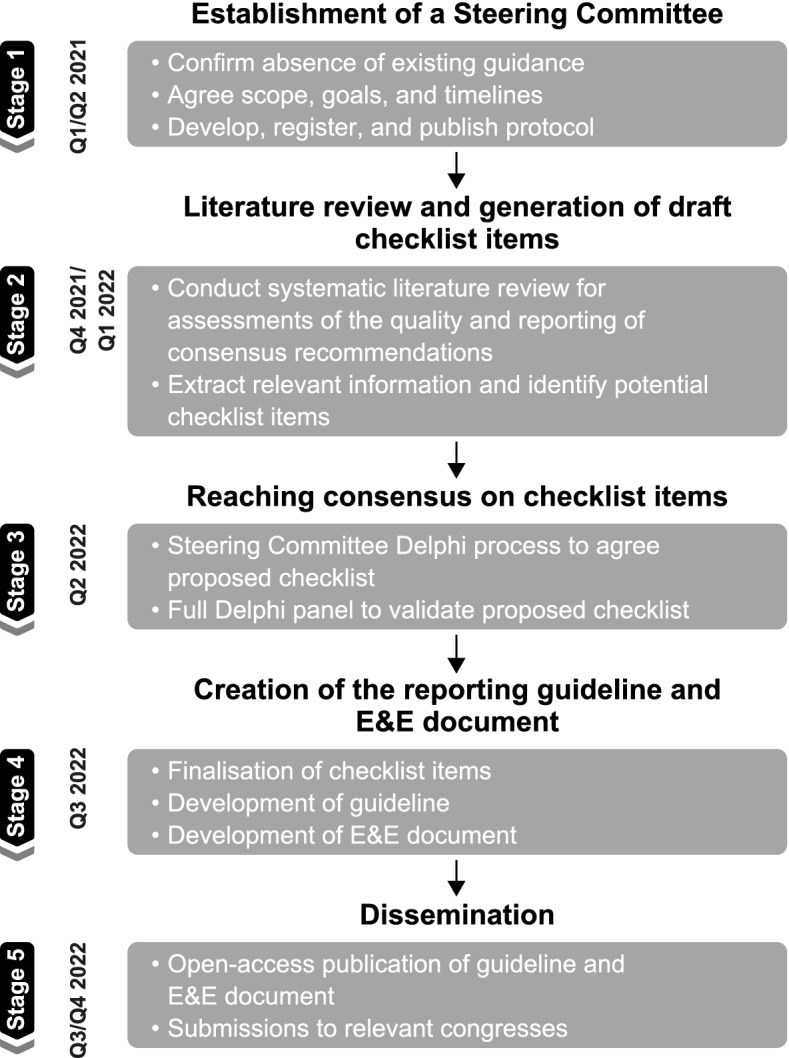
Project overview for creating ACCORD, a reporting guideline for studies developed using consensus methods

We have adopted the general method proposed by the EQUATOR Network for developing reporting guidelines [11]. The process for ACCORD development is outlined in Fig. 2.

### Stage 1: establishment of a Steering Committee

With the endorsement of the International Society of Medical Publication Professionals (ISMPP), we assembled a Steering Committee to develop a reporting guideline for research using consensus. The Steering Committee (the authors, AH, AP, CW, DT, EH, EvZ, KG, NH, PL, RM, and WG) will lead and co-ordinate the guideline development process. Specifically, the Steering Committee will be responsible for: establishing the goals and timelines for the work, including registering and publishing the protocol; generating the initial list of checklist items from the literature review; conducting a consensus process to enrich and refine the initial list of minimum items that should be reported; implementing each stage of the process including developing questionnaires and analysing voting outcomes and other data; reporting the findings of the process in a statement document with the main checklist and guidance; developing an E&E document where all the items are individually explained and examples of approach and reporting are given; disseminating the reporting guidelines via publication, presentation at congresses and other events, and online presence including a website linked to social media channels.

The Steering Committee is a multidisciplinary group (11 people) that includes clinician practitioners, methodologists, publication professionals, patients, journal editors and publishers and the pharmaceutical industry. Prior to initiating Stage 2, we listed the project in the EQUATOR Network registry for reporting guidelines under development [50] and registered the protocol with the Open Science Framework [51].

### Stage 2: literature review and generation of draft checklist items

The aim of this step is to seek evidence on the quality of reporting of the process undertaken in health studies using consensus methodology. This research will provide insight into possible checklist items for evaluation by the Delphi Panel (further information on the Delphi Panel is provided in ‘Stage 3’ below). The CREDES guidelines, specific to palliative care, will also be reviewed for elements that can be generalised to other biomedical fields [38].

#### Search strategy

The process for conducting the systematic review will be informed by and reported according to the Preferred Reporting Items for Systematic reviews and Meta-Analyses (PRISMA) 2020 and PRISMA-Search extension guidelines [11, 52]. Eligible studies will include studies, reviews and published guidance addressing the quality of reporting of consensus methodology that aim to improve health outcomes in biomedicine or clinical practice. Reports of studies using consensus methods but not commenting on their reporting quality will be excluded, for example, studies to reach clinical recommendations of core outcome sets or reporting guidelines using consensus methods. Ineligible publications include editorials, letters about individual publications, and comments on methodology of consensus outside the scope of biomedical research.

Searches of EMBASE (OVID), MEDLINE (OVID), Web of Science - Core Collection, MEDLINE (Web of Science), PubMed, Cochrane Library, and Emcare (OVID), Academic Search Premier and PsycINFO databases will be run with no limits by year or language of publication at the search stage. Four initial search strategies were developed and sequentially piloted by members of the Steering Committee (WG, EvZ and PL) with the assistance of an information (JS) and systematic review specialist (ZF). The piloting allowed the adjustment of the initial search strategy by the information specialist to provide results that better aligned with the inclusion criteria and objective of this study. The refined, broad search strategy (Supplementary File) will be used to identify and generate the final list of studies focusing on the quality and accuracy of reporting of Delphi and other consensus processes, methods, techniques or recommendations. The search may also be augmented with relevant articles highlighted by the Steering Committee as appropriate based on the individuals’ prior work and expertise in the area (via a manual search).

#### Data extraction

EvZ, PL, WG, and ZF will independently screen the titles and abstracts retrieved from the search for potential inclusion using the Rayyan tool in blind mode [53]. Any discrepancies will be resolved by discussion. Full-text articles will then be retrieved and assessed independently for eligibility, with reconciliation of any differences through discussion. Data will be extracted using a draft extraction form, which will be piloted on three studies before use. Based on the information gathered on the literature review, a list of preliminary items for the checklist will be generated to be refined in a Delphi exercise in Stage 3.

### Stage 3: reaching consensus on checklist items

**Fig. 3:**
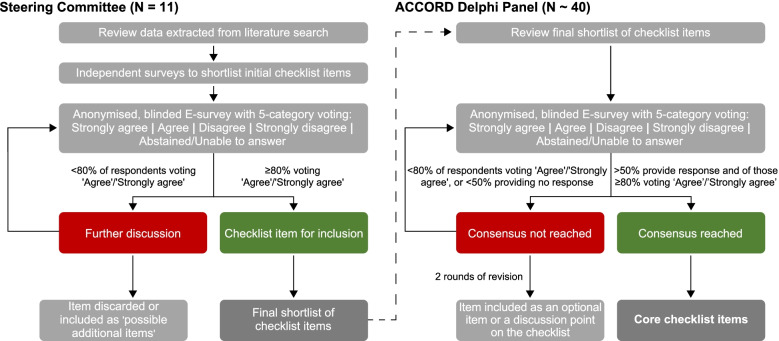
Methodology used by the ACCORD Steering Committee and ACCORD Delphi Panel to achieve consensus on core checklist items for a consensus reporting guideline

We will use Delphi methodology, as described below, to reach a consensus regarding the checklist items to include in the reporting guideline. This will take place in two steps, with the first involving the Steering Committee and the second involving a full Delphi Panel (the ACCORD Delphi Panel; Fig. 3). We plan to report the consensus methodology in accordance with our own guidelines under development.

#### First step: steering committee survey

The Steering Committee will review the data extracted from literature search. This initial list is likely to contain duplicated items or items that require rewording. The aim is to eliminate repetitions and inadequately or ambiguously written items to reach a list of unique items. Using a survey, the Steering Committee members involved in the literature review will independently suggest items for the initial checklist; NH and WG will consolidate the initial checklist items.

There will then be anonymous voting to confirm the initial checklist that will be put to the full ACCORD consensus panel. Steering Committee members (excluding NH and WG) will vote (anonymised and blinded) on whether they ‘Strongly Agree’, ‘Agree’, ‘Disagree’, ‘Strongly Disagree’, or feel ‘Abstained/Unable to answer’ for all proposed items. There will also be the opportunity to provide comments. Any items that do not receive support will be discussed by the Steering Committee, and either included as ‘possible additional items’ or discarded completely. The eliminated items and the reasons for their elimination will be reported. The candidate items will be presented in sequence as a draft checklist, and in the same order to all people voting, so that the overall checklist structure, considering the manuscript sections (such as Introduction, Methods, Results, Discussion) can be evaluated. Within each section, there will be ‘proposed items’ and ‘possible additional items’.

#### Second step: ACCORD Delphi panel

The preliminary list of checklist items agreed on by the Steering Committee will subsequently be put to the ACCORD Delphi Panel for validation using a blinded electronic voting platform (e-survey). In addition, the ACCORD Delphi Panel will be provided with the list of items excluded by the Steering Committee for information, as a confirmatory step.

The order of the candidate items within each manuscript section will be randomised so that it is different for each person voting and all items are evaluated fully independently from each other. Five voting options will be offered: ‘Strongly Agree’, ‘Agree’, ‘Disagree’, ‘Strongly Disagree’, and ‘Abstained/Unable to answer’. Votes of ‘Abstained/Unable to answer’ will be included in the denominator. Panellists will be able to provide free-text comments and will have the opportunity to propose additional items. There will be three rounds of voting; with feedback and descriptive statistics incorporated for the next round by NH and WG. The approval rate and the reasons for elimination of items will be reported.

The consensus threshold is defined in this step as at least 20 respondents (approximately 50% of the target panel size), and at least 80% of responding ACCORD Delphi panellists who are able to answer voting ‘Agree’ or ‘Strongly Agree’, with two rounds of statement revision and re-voting. The Steering Committee will review items that do not achieve consensus in rounds 1 or 2 and these will be revised or eliminated taking into account any free-text comments. If consensus is not achieved by the ACCORD Delphi Panel, or there are insufficient respondents, the Steering Committee may decide that the item will be included as an optional item or a discussion point on the E&E document or checklist, alongside core items on which consensus was achieved. Simple descriptive statistics (response rates, level of agreement for each statement, median levels of agreement and interquartile ranges) will be used to describe approval rates between rounds. The same measures will be used to evaluate consensus stability across rounds [54].

There are no generally agreed standards for the panel size for Delphi studies, and a wide range of panel sizes has been reported; panels of 20–30 participants are common [55, 56]. However, it is recognised that the size and diversity of a Delphi panel can impact the quality of the final recommendations [57]. The ACCORD Delphi Panel will comprise approximately 40 members, so that it allows for representation from clinicians, methodologists, patient advocates, lay public representatives, health technologists, journal editors and publishers, regulatory specialists, and publications professionals, and to ensure an acceptable number of responses (20, or at least 50% of the group) in the event of drop-outs or partial completion of review. The ACCORD project will be advertised to potential Delphi Panellists via relevant societies, organisations, and networks; in addition, authors of recently published consensus studies in high-profile journals will be invited directly.

When registering, panellists will be asked to complete a preliminary survey to capture basic information on experience, geographical, and demographic representation. Although no formal targets will be established, the Steering Committee will endeavour to ensure a broad spread of representation across these categories. Members of the Delphi Panel will be recognised as contributors in the acknowledgements section of the guideline (with their permission) but participation in ACCORD Delphi panel will not qualify a panellist for authorship.

Software or a voting platform that is appropriate for Delphi exercises will be used to implement the voting process, administered by NH and WG. Alternatives available on the market are being evaluated and tested at the time of this protocol publication, and the platform and version used will be reported. Initial requirements are that the software used follows security regulations, ethical standards and allows, besides voting, the inclusion of free text responses in the e-surveys to supplement discussion in the E&E document.

### Stage 4: creation of the reporting guideline and E&E document

On completion of the Delphi consensus process, the checklist will be finalised by WG and NH for approval by the Steering Committee, and the reporting guideline will be developed. A separate E&E document will be created to provide a detailed rationale for the items included in the checklist. In each case, an example will be included of good reporting from a published paper. The E&E document can also be informed by perspectives collected from researchers involved in consensus-based studies outside the biomedical field.

### Stage 5: dissemination

We intend to publish the reporting guideline and E&E document in open access format via a CC-BY copyright licence. Future publications from the ACCORD project will be reported according to the best available reporting guidelines for each type of manuscript. To aid dissemination, we plan to present the findings at congresses including ISMPP European and Annual Meetings, the World Conference on Research Integrity and Peer Review, and the UK Research Integrity Office Annual Conference. Progress will be updated on a dedicated website for the ACCORD project, the EQUATOR website and newsletter, and social media channels, and communicated in appropriate professional forums and events. This dissemination of the reporting guideline is crucial for the document to be implemented in practice.

## Discussion

The ACCORD reporting guideline will provide a set of minimum items that should be reported about methods used to achieve consensus in biomedical research and guidance, including processes ranging from simple unstructured opinion gatherings to highly structured processes. The objective is to systematically develop a reporting guideline to help the biomedical research and clinical practice community describe the methods or techniques used to reach consensus in a complete, transparent, and consistent manner.

Extensions of the ACCORD reporting guideline and checklist could potentially be developed in the future to cover consensus studies in the non-biomedical sectors, with appropriate input from experts in those sectors to account for characteristics specific to each field. Our objective is to increase the completeness, transparency and consistency of the reporting of consensus methodology and, as a result, to improve the trustworthiness of recommendations developed using consensus methods. The Steering Committee welcomes enquiries from individuals interested in participating in the ACCORD Delphi Panel.

## Supplementary Information

**Additional file 1.**

## Data Availability

Anonymised aggregated data will be deposited in the Open Science Framework, where the study protocol has already been registered (https://osf.io/2rzm9). Individual responses to Delphi rounds will be deidentified at the source level by the platform used. These individual responses and approval rates can be requested to the corresponding author. The ACCORD protocol has been listed on the EQUATOR website and pre-registered with the Open Science Framework.
