# Supplementary material for: ACCORD guideline for reporting consensus-based methods in biomedical research and clinical practice: a study protocol
Source: Res Integr Peer Rev. 2022 Jun 7;7:3. doi: 10.1186/s41073-022-00122-0 (PMC9171734; doi:10.1186/s41073-022-00122-0)
Supplement: Supplementary file 1 — Additional file 1. [file 41073_2022_122_MOESM1_ESM.docx]

**The databases to be searched are:**

Journal articles: PubMed; Web of Science; MEDLINE (OVID); Embase (OVID); Cochrane Library; Emcare (OVID)

Congress abstracts: Web of Science; Embase (OVID); Cochrane Library

**Search strings:**

**Web of Science Core Collection and MEDLINE (Web of Science)**

<http://isiknowledge.com/wos>

((TI=("Delphi Technique" OR "Delphi Technique" OR "Delphi techniques" OR "Delphi method" OR "Delphi methods" OR "Delphi study" OR "Delphi studies" OR "Delphi survey" OR "Delphi surveys" OR "Delphi consensus" OR "Delphi based consensus" OR "Delphi questionnaire" OR "Delphi questionnaires" OR "Delphi research" OR "Delphi review" OR "Delphi reviews" OR "Delphi process" OR "Delphi processes" OR "Delphi based" OR "Delphi procedure" OR "Delphi procedures" OR "Delphi assessment" OR "Delphi assessments" OR "Delphi approach" OR "Delphi approaches" OR "Delphi panel" OR "Delphi panels" OR "Delphi round" OR "Delphi rounds" OR "Delphi analysis" OR "Delphi expert" OR "Delphi experts" OR "Delphi consultation" OR "Delphi methodology" OR "nominal group technique" OR "nominal group techniques" OR "nominal group" OR "nominal groups" OR "nominal grouping" OR "consensus recommendation" OR "consensus recommendations" OR "consensus development" OR "consensus activity" OR "consensus activities" OR "Consensus Development Conference" OR "Consensus Development" OR "Consensus methodology" OR "consensus method*" OR "RAND" OR (("Guidelines" OR "guideline") NEAR/2 ("consensus" OR "delphi"))) OR AB=("Delphi Technique" OR "Delphi Technique" OR "Delphi techniques" OR "Delphi method" OR "Delphi methods" OR "Delphi study" OR "Delphi studies" OR "Delphi survey" OR "Delphi surveys" OR "Delphi consensus" OR "Delphi based consensus" OR "Delphi questionnaire" OR "Delphi questionnaires" OR "Delphi research" OR "Delphi review" OR "Delphi reviews" OR "Delphi process" OR "Delphi processes" OR "Delphi based" OR "Delphi procedure" OR "Delphi procedures" OR "Delphi assessment" OR "Delphi assessments" OR "Delphi approach" OR "Delphi approaches" OR "Delphi panel" OR "Delphi panels" OR "Delphi round" OR "Delphi rounds" OR "Delphi analysis" OR "Delphi expert" OR "Delphi experts" OR "Delphi consultation" OR "Delphi methodology" OR "nominal group technique" OR "nominal group techniques" OR "nominal group" OR "nominal groups" OR "nominal grouping" OR "consensus recommendation" OR "consensus recommendations" OR "consensus development" OR "consensus activity" OR "consensus activities" OR "Consensus Development Conference" OR "Consensus Development" OR "Consensus methodology" OR "consensus method*" OR "RAND") OR AK=("Delphi Technique" OR "Delphi Technique" OR "Delphi techniques" OR "Delphi method" OR "Delphi methods" OR "Delphi study" OR "Delphi studies" OR "Delphi survey" OR "Delphi surveys" OR "Delphi consensus" OR "Delphi based consensus" OR "Delphi questionnaire" OR "Delphi questionnaires" OR "Delphi research" OR "Delphi review" OR "Delphi reviews" OR "Delphi process" OR "Delphi processes" OR "Delphi based" OR "Delphi procedure" OR "Delphi procedures" OR "Delphi assessment" OR "Delphi assessments" OR "Delphi approach" OR "Delphi approaches" OR "Delphi panel" OR "Delphi panels" OR "Delphi round" OR "Delphi rounds" OR "Delphi analysis" OR "Delphi expert" OR "Delphi experts" OR "Delphi consultation" OR "Delphi methodology" OR "nominal group technique" OR "nominal group techniques" OR "nominal group" OR "nominal groups" OR "nominal grouping" OR "consensus recommendation" OR "consensus recommendations" OR "consensus development" OR "consensus activity" OR "consensus activities" OR "Consensus Development Conference" OR "Consensus Development" OR "Consensus methodology" OR "consensus method*" OR "RAND" OR (("Guidelines" OR "guideline") NEAR/2 ("consensus" OR "delphi")))) AND (TI=("quality of reporting" OR "reporting quality" OR "reporting qualities" OR "selective reporting" OR "poor reporting" OR "poor reported" OR "reporting guideline" OR "reporting" OR ("reporting" AND ("quality" OR "selective" OR "poor" OR "weak" OR "manner" OR "rigor" OR "improv*")) OR "Data Accuracy" OR "quality assessment" OR "strengths" OR "strength" OR "weaknesses" OR "weakness" OR "research method" OR "research methods" OR "research method*") OR AK=("quality of reporting" OR "reporting quality" OR "reporting qualities" OR "selective reporting" OR "poor reporting" OR "poor reported" OR ("reporting" NEAR/5 ("quality" OR "selective" OR "poor" OR "weak" OR "manner" OR "rigor" OR "improv*")) OR "Data Accuracy" OR "Research Report standards" OR "quality assessment" OR "strengths" OR "strength" OR "weaknesses" OR "weakness" OR "research method" OR "research methods" OR "research method*") OR AB=("quality of reporting" OR "reporting quality" OR "reporting qualities" OR "selective reporting" OR "poor reporting" OR "poor reported" OR ("reporting" NEAR/5 ("quality" OR "selective" OR "poor" OR "weak" OR "manner" OR "rigor" OR "improv*")) OR "Data Accuracy" OR "Research Report standards" OR "quality assessment" OR "strengths" OR "strength" OR "weaknesses" OR "weakness")))

**PubMed**

<http://www.ncbi.nlm.nih.gov/pubmed?otool=leiden>

(("Delphi Technique"[majr] OR "Delphi Technique"[ti] OR "Delphi techniques"[ti] OR "Delphi method"[ti] OR "Delphi methods"[ti] OR "Delphi study"[ti] OR "Delphi studies"[ti] OR "Delphi survey"[ti] OR "Delphi surveys"[ti] OR "Delphi consensus"[ti] OR "Delphi based consensus"[ti] OR "Delphi questionnaire"[ti] OR "Delphi questionnaires"[ti] OR "Delphi research"[ti] OR "Delphi review"[ti] OR "Delphi reviews"[ti] OR "Delphi process"[ti] OR "Delphi processes"[ti] OR "Delphi based"[ti] OR "Delphi procedure"[ti] OR "Delphi procedures"[ti] OR "Delphi assessment"[ti] OR "Delphi assessments"[ti] OR "Delphi approach"[ti] OR "Delphi approaches"[ti] OR "Delphi panel"[ti] OR "Delphi panels"[ti] OR "Delphi round"[ti] OR "Delphi rounds"[ti] OR "Delphi analysis"[ti] OR "Delphi expert"[ti] OR "Delphi experts"[ti] OR "Delphi consultation"[ti] OR "Delphi methodology"[ti] OR "nominal group technique"[ti] OR "nominal group techniques"[ti] OR "nominal group"[ti] OR "nominal groups"[ti] OR "nominal grouping"[ti] OR "consensus recommendation"[ti] OR "consensus recommendations"[ti] OR "consensus development"[ti] OR "consensus activity"[ti] OR "consensus activities"[ti] OR "consensus methodology"[ti] OR "consensus method*"[ti] OR "Consensus Development Conferences as Topic"[majr] OR "RAND"[ti] OR ("Guidelines as Topic"[majr:noexp] AND ("consensus"[tw] OR "delphi"[tw]))) AND ("reporting"[ti] OR "quality of reporting"[tw] OR "reporting quality"[tw] OR "reporting qualities"[tw] OR "selective reporting"[tw] OR "poor reporting"[tw] OR "poor reported"[tw] OR "poorly reported"[tw] OR "Research Report/standards"[majr] OR "Research Design/standards"[mesh] OR "Research Design"[majr:noexp] OR "Writing/standards"[mesh] OR "Writing"[majr] OR "research method"[ti] OR "research methods"[ti] OR "research method*"[ti]))

**MEDLINE via OVID**

<http://gateway.ovid.com/ovidweb.cgi?T=JS&MODE=ovid&NEWS=n&PAGE=main&D=medall>

((exp *"Delphi Technique"/ OR "Delphi Technique".ti OR "Delphi techniques".ti OR "Delphi method".ti OR "Delphi methods".ti OR "Delphi study".ti OR "Delphi studies".ti OR "Delphi survey".ti OR "Delphi surveys".ti OR "Delphi consensus".ti OR "Delphi based consensus".ti OR "Delphi questionnaire".ti OR "Delphi questionnaires".ti OR "Delphi research".ti OR "Delphi review".ti OR "Delphi reviews".ti OR "Delphi process".ti OR "Delphi processes".ti OR "Delphi based".ti OR "D

elphi procedure".ti OR "Delphi procedures".ti OR "Delphi assessment".ti OR "Delphi assessments".ti OR "Delphi approach".ti OR "Delphi approaches".ti OR "Delphi panel".ti OR "Delphi panels".ti OR "Delphi round".ti OR "Delphi rounds".ti OR "Delphi analysis".ti OR "Delphi expert".ti OR "Delphi experts".ti OR "Delphi consultation".ti OR "Delphi methodology".ti OR "nominal group technique".ti OR "nominal group techniques".ti OR "nominal group".ti OR "nominal groups".ti OR "nominal grouping".ti OR "consensus recommendation".ti OR "consensus recommendations".ti OR "consensus development".ti OR "consensus activity".ti OR "consensus activities".ti OR "consensus methodology".ti OR "consensus method*".ti OR exp *"Consensus Development Conferences as Topic"/ OR "RAND".ti OR ("Guidelines as Topic"/ AND ("consensus".mp OR "delphi".mp)) OR (("Guidelines".mp OR "guideline".mp) ADJ2 ("consensus".mp OR "delphi".mp))) AND ("reporting".ti OR "quality of reporting".mp OR "reporting quality".mp OR "reporting qualities".mp OR "selective reporting".mp OR "poor reporting".mp OR "poor reported".mp OR "poorly reported".mp OR "Research Report/standards"/ OR exp "Research Design"/st OR *"Research Design"/ OR exp "Writing"/st OR exp *"Writing"/ OR "research method".ti OR "research methods".ti OR "research method*".ti OR ("reporting" ADJ8 ("quality" OR "selective" OR "poor" OR "weak" OR "manner" OR "rigor" OR "improv*")).mp))

**Embase**

<http://ovidsp.ovid.com/ovidweb.cgi?T=JS&PAGE=main&MODE=ovid&D=oemezd>

((exp *"Delphi Study"/ OR "Delphi Technique".ti OR "Delphi techniques".ti OR "Delphi method".ti OR "Delphi methods".ti OR "Delphi study".ti OR "Delphi studies".ti OR "Delphi survey".ti OR "Delphi surveys".ti OR "Delphi consensus".ti OR "Delphi based consensus".ti OR "Delphi questionnaire".ti OR "Delphi questionnaires".ti OR "Delphi research".ti OR "Delphi review".ti OR "Delphi reviews".ti OR "Delphi process".ti OR "Delphi processes".ti OR "Delphi based".ti OR "Delphi procedure".ti OR "Delphi procedures".ti OR "Delphi assessment".ti OR "Delphi assessments".ti OR "Delphi approach".ti OR "Delphi approaches".ti OR "Delphi panel".ti OR "Delphi panels".ti OR "Delphi round".ti OR "Delphi rounds".ti OR "Delphi analysis".ti OR "Delphi expert".ti OR "Delphi experts".ti OR "Delphi consultation".ti OR "Delphi methodology".ti OR "nominal group technique".ti OR "nominal group techniques".ti OR "nominal group".ti OR "nominal groups".ti OR "nominal grouping".ti OR "consensus recommendation".ti OR "consensus recommendations".ti OR "consensus development".ti OR "consensus activity".ti OR "consensus activities".ti OR "consensus methodology".ti OR "consensus method*".ti OR exp *"Consensus Development"/ OR "RAND".ti OR (("Guidelines".ti,ab OR "guideline".ti,ab) ADJ2 ("consensus".ti,ab OR "delphi".ti,ab))) AND ("reporting".ti OR "quality of reporting".ti,ab OR "reporting quality".ti,ab OR "reporting qualities".ti,ab OR "selective reporting".ti,ab OR "poor reporting".ti,ab OR "poor reported".ti,ab OR "poorly reported".ti,ab OR *"Methodology"/ OR *"Writing"/ OR "research method".ti OR "research methods".ti OR "research method*".ti OR ("reporting" ADJ8 ("quality" OR "selective" OR "poor" OR "weak" OR "manner" OR "rigor" OR "improv*")).ti,ab))

**Cochrane**

<https://www.cochranelibrary.com/advanced-search/search-manager>

(("Delphi Technique" OR "Delphi Technique" OR "Delphi techniques" OR "Delphi method" OR "Delphi methods" OR "Delphi study" OR "Delphi studies" OR "Delphi survey" OR "Delphi surveys" OR "Delphi consensus" OR "Delphi based consensus" OR "Delphi questionnaire" OR "Delphi questionnaires" OR "Delphi research" OR "Delphi review" OR "Delphi reviews" OR "Delphi process" OR "Delphi processes" OR "Delphi based" OR "Delphi procedure" OR "Delphi procedures" OR "Delphi assessment" OR "Delphi assessments" OR "Delphi approach" OR "Delphi approaches" OR "Delphi panel" OR "Delphi panels" OR "Delphi round" OR "Delphi rounds" OR "Delphi analysis" OR "Delphi expert" OR "Delphi experts" OR "Delphi consultation" OR "Delphi methodology" OR "nominal group technique" OR "nominal group techniques" OR "nominal group" OR "nominal groups" OR "nominal grouping" OR "consensus recommendation" OR "consensus recommendations" OR "consensus development" OR "consensus activity" OR "consensus activities" OR "Consensus Development Conference" OR "Consensus Development" OR "Consensus methodology" OR "consensus method*" OR "RAND" OR (("Guidelines" OR "guideline") NEAR/2 ("consensus" OR "delphi"))) AND ("quality of reporting" OR "reporting quality" OR "reporting qualities" OR "selective reporting" OR "poor reporting" OR "poor reported" OR ("reporting" NEAR/5 ("quality" OR "selective" OR "poor" OR "weak" OR "manner" OR "rigor" OR "improv*")) OR "Data Accuracy" OR "Research Report standards" OR "quality assessment" OR "strengths" OR "strength" OR "weaknesses" OR "weakness" OR "research method" OR "research methods" OR "research method*")):ti,ab,kw

**Emcare** <http://ovidsp.ovid.com/ovidweb.cgi?T=JS&NEWS=n&CSC=Y&PAGE=main&D=emcr>

((exp *"Delphi Study"/ OR "Delphi Technique".ti OR "Delphi techniques".ti OR "Delphi method".ti OR "Delphi methods".ti OR "Delphi study".ti OR "Delphi studies".ti OR "Delphi survey".ti OR "Delphi surveys".ti OR "Delphi consensus".ti OR "Delphi based consensus".ti OR "Delphi questionnaire".ti OR "Delphi questionnaires".ti OR "Delphi research".ti OR "Delphi review".ti OR "Delphi reviews".ti OR "Delphi process".ti OR "Delphi processes".ti OR "Delphi based".ti OR "Delphi procedure".ti OR "Delphi procedures".ti OR "Delphi assessment".ti OR "Delphi assessments".ti OR "Delphi approach".ti OR "Delphi approaches".ti OR "Delphi panel".ti OR "Delphi panels".ti OR "Delphi round".ti OR "Delphi rounds".ti OR "Delphi analysis".ti OR "Delphi expert".ti OR "Delphi experts".ti OR "Delphi consultation".ti OR "Delphi methodology".ti OR "nominal group technique".ti OR "nominal group techniques".ti OR "nominal group".ti OR "nominal groups".ti OR "nominal grouping".ti OR "consensus recommendation".ti OR "consensus recommendations".ti OR "consensus development".ti OR "consensus activity".ti OR "consensus activities".ti OR "consensus methodology".ti OR "consensus method*".ti OR exp *"Consensus Development"/ OR "RAND".ti OR (("Guidelines".ti,ab OR "guideline".ti,ab) ADJ2 ("consensus".ti,ab OR "delphi".ti,ab))) AND ("reporting".ti OR "quality of reporting".ti,ab OR "reporting quality".ti,ab OR "reporting qualities".ti,ab OR "selective reporting".ti,ab OR "poor reporting".ti,ab OR "poor reported".ti,ab OR "poorly reported".ti,ab OR *"Methodology"/ OR *"Writing"/ OR "research method".ti OR "research methods".ti OR "research method*".ti OR ("reporting" ADJ8 ("quality" OR "selective" OR "poor" OR "weak" OR "manner" OR "rigor" OR "improv*")).ti,ab))

**Academic Search Premier**

<http://search.ebscohost.com/login.aspx?authtype=ip,uid&profile=lumc&defaultdb=aph>

((TI("Delphi Technique" OR "Delphi Technique" OR "Delphi techniques" OR "Delphi method" OR "Delphi methods" OR "Delphi study" OR "Delphi studies" OR "Delphi survey" OR "Delphi surveys" OR "Delphi consensus" OR "Delphi based consensus" OR "Delphi questionnaire" OR "Delphi questionnaires" OR "Delphi research" OR "Delphi review" OR "Delphi reviews" OR "Delphi process" OR "Delphi processes" OR "Delphi based" OR "Delphi procedure" OR "Delphi procedures" OR "Delphi assessment" OR "Delphi assessments" OR "Delphi approach" OR "Delphi approaches" OR "Delphi panel" OR "Delphi panels" OR "Delphi round" OR "Delphi rounds" OR "Delphi analysis" OR "Delphi expert" OR "Delphi experts" OR "Delphi consultation" OR "Delphi methodology" OR "nominal group technique" OR "nominal group techniques" OR "nominal group" OR "nominal groups" OR "nominal grouping" OR "consensus recommendation" OR "consensus recommendations" OR "consensus development" OR "consensus activity" OR "consensus activities" OR "Consensus Development Conference" OR "Consensus Development" OR "Consensus methodology" OR "consensus method*" OR "RAND" OR (("Guidelines" OR "guideline") N2 ("consensus" OR "delphi"))) OR KW("Delphi Technique" OR "Delphi Technique" OR "Delphi techniques" OR "Delphi method" OR "Delphi methods" OR "Delphi study" OR "Delphi studies" OR "Delphi survey" OR "Delphi surveys" OR "Delphi consensus" OR "Delphi based consensus" OR "Delphi questionnaire" OR "Delphi questionnaires" OR "Delphi research" OR "Delphi review" OR "Delphi reviews" OR "Delphi process" OR "Delphi processes" OR "Delphi based" OR "Delphi procedure" OR "Delphi procedures" OR "Delphi assessment" OR "Delphi assessments" OR "Delphi approach" OR "Delphi approaches" OR "Delphi panel" OR "Delphi panels" OR "Delphi round" OR "Delphi rounds" OR "Delphi analysis" OR "Delphi expert" OR "Delphi experts" OR "Delphi consultation" OR "Delphi methodology" OR "nominal group technique" OR "nominal group techniques" OR "nominal group" OR "nominal groups" OR "nominal grouping" OR "consensus recommendation" OR "consensus recommendations" OR "consensus development" OR "consensus activity" OR "consensus activities" OR "Consensus Development Conference" OR "Consensus Development" OR "Consensus methodology" OR "consensus method*" OR "RAND" OR (("Guidelines" OR "guideline") N2 ("consensus" OR "delphi")))) AND (TI("quality of reporting" OR "reporting quality" OR "reporting qualities" OR "selective reporting" OR "poor reporting" OR "poor reported" OR "reporting guideline" OR "reporting" OR ("reporting" AND ("quality" OR "selective" OR "poor" OR "weak" OR "manner" OR "rigor" OR "improv*")) OR "Data Accuracy" OR "quality assessment" OR "strengths" OR "strength" OR "weaknesses" OR "weakness" OR "research method" OR "research methods" OR "research method*") OR KW("quality of reporting" OR "reporting quality" OR "reporting qualities" OR "selective reporting" OR "poor reporting" OR "poor reported" OR ("reporting" N5 ("quality" OR "selective" OR "poor" OR "weak" OR "manner" OR "rigor" OR "improv*")) OR "Data Accuracy" OR "Research Report standards" OR "quality assessment" OR "strengths" OR "strength" OR "weaknesses" OR "weakness" OR "research method" OR "research methods" OR "research method*") OR AB("quality of reporting" OR "reporting quality" OR "reporting qualities" OR "selective reporting" OR "poor reporting" OR "poor reported" OR ("reporting" N5 ("quality" OR "selective" OR "poor" OR "weak" OR "manner" OR "rigor" OR "improv*")) OR "Data Accuracy" OR "Research Report standards" OR "quality assessment" OR "strengths" OR "strength" OR "weaknesses" OR "weakness")))

**PsycINFO**

<http://search.ebscohost.com/login.aspx?authtype=ip,uid&profile=lumc&defaultdb=psyh>

((TI("Delphi Technique" OR "Delphi Technique" OR "Delphi techniques" OR "Delphi method" OR "Delphi methods" OR "Delphi study" OR "Delphi studies" OR "Delphi survey" OR "Delphi surveys" OR "Delphi consensus" OR "Delphi based consensus" OR "Delphi questionnaire" OR "Delphi questionnaires" OR "Delphi research" OR "Delphi review" OR "Delphi reviews" OR "Delphi process" OR "Delphi processes" OR "Delphi based" OR "Delphi procedure" OR "Delphi procedures" OR "Delphi assessment" OR "Delphi assessments" OR "Delphi approach" OR "Delphi approaches" OR "Delphi panel" OR "Delphi panels" OR "Delphi round" OR "Delphi rounds" OR "Delphi analysis" OR "Delphi expert" OR "Delphi experts" OR "Delphi consultation" OR "Delphi methodology" OR "nominal group technique" OR "nominal group techniques" OR "nominal group" OR "nominal groups" OR "nominal grouping" OR "consensus recommendation" OR "consensus recommendations" OR "consensus development" OR "consensus activity" OR "consensus activities" OR "Consensus Development Conference" OR "Consensus Development" OR "Consensus methodology" OR "consensus method*" OR "RAND" OR (("Guidelines" OR "guideline") N2 ("consensus" OR "delphi"))) OR AB("Delphi Technique" OR "Delphi Technique" OR "Delphi techniques" OR "Delphi method" OR "Delphi methods" OR "Delphi study" OR "Delphi studies" OR "Delphi survey" OR "Delphi surveys" OR "Delphi consensus" OR "Delphi based consensus" OR "Delphi questionnaire" OR "Delphi questionnaires" OR "Delphi research" OR "Delphi review" OR "Delphi reviews" OR "Delphi process" OR "Delphi processes" OR "Delphi based" OR "Delphi procedure" OR "Delphi procedures" OR "Delphi assessment" OR "Delphi assessments" OR "Delphi approach" OR "Delphi approaches" OR "Delphi panel" OR "Delphi panels" OR "Delphi round" OR "Delphi rounds" OR "Delphi analysis" OR "Delphi expert" OR "Delphi experts" OR "Delphi consultation" OR "Delphi methodology" OR "nominal group technique" OR "nominal group techniques" OR "nominal group" OR "nominal groups" OR "nominal grouping" OR "consensus recommendation" OR "consensus recommendations" OR "consensus development" OR "consensus activity" OR "consensus activities" OR "Consensus Development Conference" OR "Consensus Development" OR "Consensus methodology" OR "consensus method*" OR "RAND") OR KW("Delphi Technique" OR "Delphi Technique" OR "Delphi techniques" OR "Delphi method" OR "Delphi methods" OR "Delphi study" OR "Delphi studies" OR "Delphi survey" OR "Delphi surveys" OR "Delphi consensus" OR "Delphi based consensus" OR "Delphi questionnaire" OR "Delphi questionnaires" OR "Delphi research" OR "Delphi review" OR "Delphi reviews" OR "Delphi process" OR "Delphi processes" OR "Delphi based" OR "Delphi procedure" OR "Delphi procedures" OR "Delphi assessment" OR "Delphi assessments" OR "Delphi approach" OR "Delphi approaches" OR "Delphi panel" OR "Delphi panels" OR "Delphi round" OR "Delphi rounds" OR "Delphi analysis" OR "Delphi expert" OR "Delphi experts" OR "Delphi consultation" OR "Delphi methodology" OR "nominal group technique" OR "nominal group techniques" OR "nominal group" OR "nominal groups" OR "nominal grouping" OR "consensus recommendation" OR "consensus recommendations" OR "consensus development" OR "consensus activity" OR "consensus activities" OR "Consensus Development Conference" OR "Consensus Development" OR "Consensus methodology" OR "consensus method*" OR "RAND" OR (("Guidelines" OR "guideline") N2 ("consensus" OR "delphi")))) AND (TI("quality of reporting" OR "reporting quality" OR "reporting qualities" OR "selective reporting" OR "poor reporting" OR "poor reported" OR "reporting guideline" OR "reporting" OR ("reporting" AND ("quality" OR "selective" OR "poor" OR "weak" OR "manner" OR "rigor" OR "improv*")) OR "Data Accuracy" OR "quality assessment" OR "strengths" OR "strength" OR "weaknesses" OR "weakness" OR "research method" OR "research methods" OR "research method*") OR KW("quality of reporting" OR "reporting quality" OR "reporting qualities" OR "selective reporting" OR "poor reporting" OR "poor reported" OR ("reporting" N5 ("quality" OR "selective" OR "poor" OR "weak" OR "manner" OR "rigor" OR "improv*")) OR "Data Accuracy" OR "Research Report standards" OR "quality assessment" OR "strengths" OR "strength" OR "weaknesses" OR "weakness" OR "research method" OR "research methods" OR "research method*") OR AB("quality of reporting" OR "reporting quality" OR "reporting qualities" OR "selective reporting" OR "poor reporting" OR "poor reported" OR ("reporting" N5 ("quality" OR "selective" OR "poor" OR "weak" OR "manner" OR "rigor" OR "improv*")) OR "Data Accuracy" OR "Research Report standards" OR "quality assessment" OR "strengths" OR "strength" OR "weaknesses" OR "weakness")))
